# Spicule formation in calcareous sponges: Coordinated expression of biomineralization genes and spicule-type specific genes

**DOI:** 10.1038/srep45658

**Published:** 2017-04-13

**Authors:** Oliver Voigt, Maja Adamska, Marcin Adamski, André Kittelmann, Lukardis Wencker, Gert Wörheide

**Affiliations:** 1 Department of Earth and Environmental Sciences, Palaeontology and Geobiology, Ludwig-Maximilians-Universität München, Richard-Wagner-Str. 10, 80333 Munich, Germany; 2 Research School of Biology, ANU College of Medicine, Biology and Environment, The Australian National University, Canberra, 46 Sullivans Creek Road, Acton ACT 2601, Australia; 3 GeoBio-Center, Ludwig-Maximilians-Universität München, Richard-Wagner-Str. 10, 80333 München, Germany; 4 Bayerische Staatssammlung für Paläontologie und Geologie, Richard-Wagner-Str. 10, 80333 München, Germany

## Abstract

The ability to form mineral structures under biological control is widespread among animals. In several species, specific proteins have been shown to be involved in biomineralization, but it is uncertain how they influence the shape of the growing biomineral and the resulting skeleton. Calcareous sponges are the only sponges that form calcitic spicules, which, based on the number of rays (actines) are distinguished in diactines, triactines and tetractines. Each actine is formed by only two cells, called sclerocytes. Little is known about biomineralization proteins in calcareous sponges, other than that specific carbonic anhydrases (CAs) have been identified, and that uncharacterized Asx-rich proteins have been isolated from calcitic spicules. By RNA-Seq and RNA *in situ* hybridization (ISH), we identified five additional biomineralization genes in *Sycon ciliatum:* two bicarbonate transporters (BCTs) and three Asx-rich extracellular matrix proteins (ARPs). We show that these biomineralization genes are expressed in a coordinated pattern during spicule formation. Furthermore, two of the ARPs are spicule-type specific for triactines and tetractines (ARP1 or *SciTriactinin*) or diactines (ARP2 or *SciDiactinin*). Our results suggest that spicule formation is controlled by defined temporal and spatial expression of spicule-type specific sets of biomineralization genes.

By the process of biomineralization many animal groups produce mineral structures like skeletons, shells and teeth. Biominerals differ in shape considerably from their inorganic mineral counterparts^1^. In order to build specific skeletal structures, organisms have to control the biomineralization process. This control involves proteins with different functions. For calcium carbonate biominerals, which are the most widespread minerals formed by animals^2^, the directional transport and accumulation of inorganic ions to the calcification site is achieved by specialized transporters, i.e. by bicarbonate transporters (BCTs) or Ca^2+^-transporters e.g., refs 3 and 4. Linked to bicarbonate transport and pH-regulation is the catalytic activity of carbonic anhydrases (CAs), which catalyse the reversible reaction of CO_2_ to bicarbonate^5^. Specialized CAs are key biomineralization proteins in calcium carbonate producing animals^6^. In addition, proteins of the skeletal organic matrix (SOM) have been identified by means of proteomics, transcriptomics and genomics^7^. Skeletal proteomes comprise mostly secreted proteins, and often include acidic proteins with high proportions of the amino acids aspartic acid or glutamic acid^7^,^8^. These acidic SOM proteins presumably interact with the calcium carbonate crystals and thereby can influence the growth and shape of biominerals^9^. However, little is known how the expression of biomineralization genes is coordinated and influences the biomineral shape.

Calcareous sponges (Porifera, class Calcarea) are an ideal system to address this question. Their calcite spicules are relatively simple structures, which can be distinguished by the number of their rays (actines) in monaxonic diactines (initially growing in two directions), three-rayed triactines, and four-rayed tetractines^10^ (Fig. 1A). They are produced by only a few specialized cells, the sclerocytes, often within just a few days^11^,^12^. The spicules are growing within an extracellular space, sealed by septate junctions between the membranes of the sclerocytes^13^,^14^, and are surrounded by an organic sheath that is secreted by the sclerocytes^14^. Each spicule is formed by two (diactines), six (triactines) or seven (tetractines) sclerocytes, of which one (termed founder cell) promotes tip growth, and the other, at least in some species, thickens the spicule (the thickener cell)^15^,^16^ (Fig. 1B,C). Each founder and thickener cell pair originates from the division of a precursor cell; in case of triactine sclerocytes, these precursors form triplets before they divide^14^,^15^,^16^. At least in diactines, based on spicule staining experiments^11^ and TEM observations^14^ it was suggested that during initial stages of spicule formation the two sclerocytes contribute equally to tip elongation, before one starts functioning as a thickener cell.

Little is known about biomineralization genes in calcareous sponges; only two specific CAs have been identified^12^,^17^,^18^. Furthermore, Asx (aspartic acid or asparagine)- rich proteins (ARPs) were extracted from spicules of different species, but have been only characterized by their amino acid composition^19^,^20^. We performed our study on the widespread calcareous sponge *Sycon ciliatum*, a model species for developmental biology with a sequenced genome^21^,^22^,^23^. The spicule formation by sclerocytes in this species has been documented by light microscopy^15^ and electron microscopy^13^,^14^. *Sycon ciliatum* has four spicule types (Fig. 1A), which can be readily distinguished and occur in specific body parts: (1) long, slender diactines (also called trichoxea), which form a palisade-like ring structure around the osculum; (2) curved diactines, which are restricted to the distal end of the radial tubes; (3) triactines, which form the atrial skeleton and the walls of the radial tubes; and (4) tetractines, which occur in the atrial skeleton (Fig. 1A). Triactines and tetractines with their three-rayed basal system form a scaffolding support for the tissues of the radial tubes (including the innermost layer of the water-propelling and filtering choanocytes), and the central cavity. Diactines, which protrude from the sponge body at the tips of the radial tubes and around the osculum, may serve as mechanical protection against blockage of influx and efflux openings.

A previous study found that spicule formation and the expression of two biomineralization genes, the carbonic anhydrases *SciCA1* and *SciCA2*, is increased in the apical part of *S. ciliatum* sponges, where new radial tubes and the slender diactines of the osculum are built^12^. By RNA-Seq analysis we identified additional key biomineralization genes of calcareous sponges and studied their temporal and spatial expression patterns by RNA *in situ hybridisation* (ISH) to understand how they interact in the spicule formation process.

## Results and Discussion

### Identification and expression patterns of biomineralization candidate genes

We identified new additional genes involved in biomineralization in *Sycon ciliatum* by screening RNA-Seq data of apically overexpressed genes^22^,^24^ for potential candidates, focussing on bicarbonate transporters and secreted, Asx-rich, proteins (ARPs). Bicarbonate transporters of the solute carrier 4 (SLC4) family are known to be involved in carbon transport and pH regulation^25^, and a specific variant has been shown to be a key biomineralization gene in scleractinian corals^4^. ARPs appear to be a major component of the spicule matrix proteome of calcareous sponges, as revealed by analyses of amino acid composition from proteins isolated from the spicules of various species^19^,^20^.

Among the apically overexpressed transcripts, we identified two SLC4 proteins and three ARPs with signal peptides (ARP1-3). Partial or complete coding sequences were PCR-amplified, cloned and sequenced. The sclerocyte-specific expression of all five genes was verified by *in situ* hybridization (ISH), confirming their expected involvement in biomineralization (Fig. 2). To further interpret the expression patterns in the absence of the calcitic spicules, which dissolve during the ISH procedure, double ISH was performed with two different colour detections with combinations of probes for the five new genes and the previously studied carbonic anhydrases SciCA1 and *SciCA2*^12^.

### Expression patterns of sclerocyte-specific *S. ciliatum* SLC4 proteins

Phylogenetic analyses (Fig. 3) of SLC4 proteins revealed that one (scigt008985) of the identified, potentially sclerocyte-specific SLC4-proteins of *S. ciliatum* belongs to the group of Na^+^/HCO_3_^−^ co-transport proteins (NCBT-like), and that the other one (scigt015021) falls in the group of HCO_3_^−^/Cl^−^ anion exchange proteins (AE-like). We therefore termed these *Sycon ciliatum* SLC4 proteins SciNCBT-like1 (scigt008985), and SciAE-like1 (scigt015021). Additional SLC4 proteins of *S.ciliatum* also belong to the two SLC4 groups and accordingly were termed *SciNCBT*-*like2* (scigt018445), *SciAE-like2* (scigt016671) and *SciAE-like3* (scigt026034).

S*ciNCBT-like1* and *SciAE-like1* showed similar expression patterns. They were expressed in founder and thickener cells of all spicule types, similar to the *S. ciliatum* carbonic anhydrase *SciCA2*^12^ (Fig. 2A–C). Both are expressed in regions of increased spicule formation and expressing cells form an oscular ring (Fig. 2B,C), and are more abundant in the upper radial tubes (Suppl. Figure 1). Expression occurred in sclerocytes of diactines, triactines and tetractines. In the latter two, expression occurred in all six cells of the initial sextet (Fig. 2B,C). Double ISH with ARP1 revealed further details (see below).

### Expression patterns and properties of ARPs

**Figure 1:**
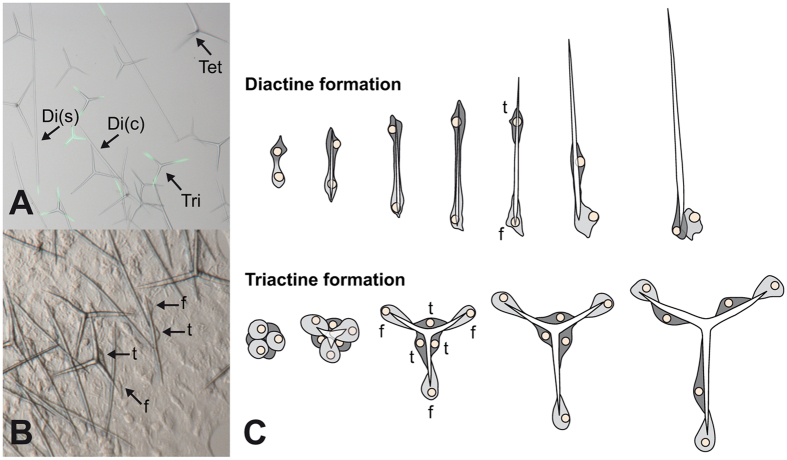
Spicule types and spicule formation in *S. ciliatum.* (**A)** Isolated spicules (fluorescence of calcein staining overlayed, showing spicule growth during 18 h^12^): Di(s) = oscular slender diactines, Di (c) = curved diactines of the distal end of radial tubes, Tri = triactines of the radial tubes and the atrial skeleton, Tet = tetractines of the atrial skeleton. (**B)** *In vivo* formation of spicules by sclerocytes (f = founder cell, t = thickener cell). (**C**) Movement of founder (f) and thickener (t) cells during diactine and triactine formation. (**A**) and (**C**) modified from ref. 12, (**C**) partially redrawn from ref. 16.

**Figure 2:**
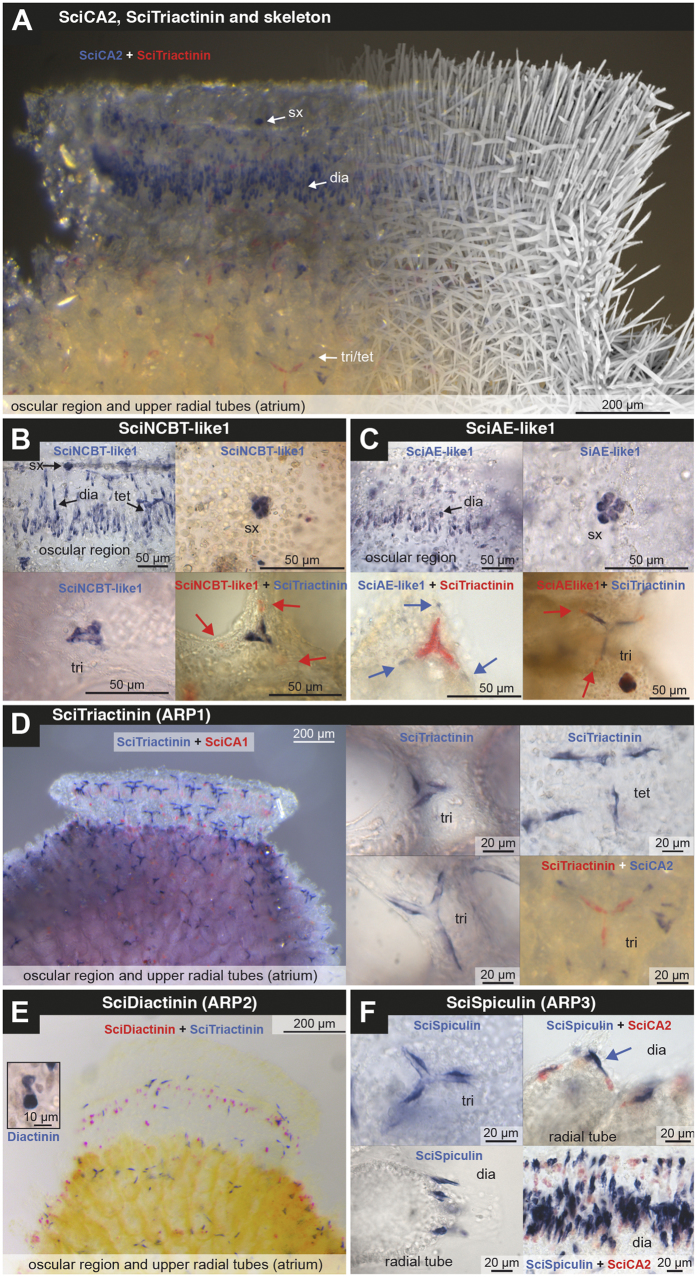
Expression patterns of biomineralization genes. (**A**) Overview over oscular region (atrial side) with *SciCA2* expression (blue), *SciTriactinin* expression (red) and an overlay of a μCT image to show the position of the dissolved spicules. (**B**) *SciNCBT-like1* expression in diactine, triactine and tetractine sclerocytes. Double-ISH with SciTriactinin-specific probes suggests expression in founder cells at later stages of spicule formation. (**C**) *SciAE-like1* expression in diactine, triactine and tetractine sclerocytes. Double-ISH with SciTriactinin-specific probes suggests expression in founder cells at later stages of spicule formation. (**D**) *SciTriactinin* expression is specific to triactine and tetractine thickener cells. *SciCA1* is only expressed in early stages, *SciTriactinin* in later stages. Double ISH with *SciCA2* reveals that at later stages of triactine and tetractine formation *SciCA2* expression only occurs in founder cells. (**E**) Expression of *SciDiactinin* in diactine forming sclerocytes. Inset: two close diactine-forming sclerocytes of early diactine formation. (**F**) *SciSpiculin* expression in thickener cells of triactines (tetractines not shown) and diactines. Double ISH with *SciCA2* reveals that founder cells of diactines are not expressing *SciSpiculin*, but *SciCA2* (radial tubes and oscular diactines). Abbreviations: dia = diactines, sx = sextet of sclerocytes, early stage of triactine (and tetractine) formation (compare Fig. 1C), tri = triactines; tet = tetractines.

In contrast to *SciNCBT-like1* and *SciAE-like1*, the expression patterns of the three ARPs were more specific: *ARP1* (scigt005329) was exclusively expressed in founder cells of tri- and tetractines (Fig. 1D); we therefore termed this protein SciTriactinin. *ARP2* (scigt017205) was expressed mostly in cells found in the oscular region, in which oscular diactines are formed, and in the distal end of radial tubes, where curved diactines are built (Fig. 1E). On several occasions, *ARP2* expression occurred in two close sclerocytes (Fig. 2E, inset). When detected together in double ISH with SciCA2, a marker of active sclerocytes^12^, only a small fraction of active sclerocytes expressed *ARP2* (Suppl. Figure 1). In our view, these ISH patterns suggest expression only in a short time during spicule formation in early-stage diactine sclerocytes. Because no expression in triactine- or tetractine-specific sclerocytes was detected, we named this protein SciDiactinin. Finally, *ARP3* (scigt005329) was expressed in thickener cells of all spicule types in later stages of spicule formation (Fig. 1F, Suppl. Figure 1). Accordingly, we termed this protein SciSpiculin, in reference to Haeckel’s name for unidentified organic components in calcareous sponge spicules^26^.

SciTriactinin, SciDiactinin and SciSpiculin are short proteins (with 143, 158 and 418 amino acids, respectively, Fig. 4), with an N-terminal signal peptide and a high content of aspartic acid, which makes them highly acidic (isoelectric points 3.6–3.8). Additionally, serine is a frequent amino acid in these proteins. Several O-linked glycosylation sites are predicted by Glyco EP^27^ in all three ARPs, but only SciTriactinin has three potential N-linked glycosylation sites. Despite a short, shared motif (ADPPTP) found near the C-terminus of SciTriactinin and SciDiactinin, the three ARPs are not particularly similar to each other. Spiculin is characterized by a 39 amino acid repeat motif, which was present in eight complete (five in the genomic sequence, see Methods), and one partial copy in the cDNA sequence (Fig. 4). Previous reports about high Asx and serine content in proteins isolated from the intraspicular matrix of several calcareous sponge species suggested that acidic proteins are a major component of the spicule matrix proteome^19^,^20^. Therefore, we propose that SciTriactinin, SciDiactinin and SciSpiculin are important intraspicular matrix proteins. This proposal is supported by (1) the higher expression of these genes in the top body part of *S. ciliatum*, where increased spicule formation occurs; (2) the sclerocyte-specific expression of the ARPs; and (3) the presence of signal peptides, and therefore their potential secretion into the extracellular space of spicule formation.

### Temporal and spatial expression of biomineralization genes during spicule formation

The expression levels in different body parts (top, middle bottom, Fig. 5A) were studied by RNA-Seq, using the available datasets^22^. The expression profiles of *SciNCBT-like1* and *SciAE-like1* were similar to that of *SciCA1* und *SciCA2*^12^ regarding their apical overexpression and maximum expression levels (Fig. 5B). Of the remaining SLC4 proteins, *SciNCBT-like2, SciAE-like2* had equal expression levels in all body parts, and expression levels of *SciAE-like3* were much lower (Fig. 5B). Maximal expression levels of the three ARPs were lower compared to the sclerocyte-specific CAs and BCTs. All were significantly higher expressed in apical parts in comparison to middle body parts, and, with exception of *SciDiactinin*, to bottom body parts.

Double ISH of combinations of biomineralization gene probes provided additional insight into the temporal and spatial expression in different stages of spicule formation: the results are summarized in Fig. 5C. *SciNCBT-like1, SciAE-like1* and *SciCA1* and *SciCA2* are expressed in all sclerocytes of all spicule types in the initial spicule formation stages (*SciCA2* expression begins later^12^). At later stages, when the founder and the thickener cells become separated, the expression of these genes is restricted to the founder cells. At this stage, we did not observe expression of *SciCA1* (Fig. 2D). The expression of *SciNCBT-like1* (Fig. 2B), *SciAE-like1* (Fig. 2C) in founder cells in these later spicule formation stages was less frequently observed than the expression of *SciCA2* (Fig. 2A,D); therefore, their expression likely ceases earlier. In the case of the SLC4 transporters, it can be assumed that these transmembrane transporters remain functional for a certain amount of time after their formation; so their production may not be necessary until the very end of the spicule growth. *SciSpiculin* is expressed in thickener cells of all spicule types in later spicule formation stages, again, after the separation of founder and thickener cell (Fig. 2F). In contrast, *SciDiactinin* and *SciTriactinin* are spicule type-specific. *SciDiactinin* is expressed in both, founder and prospective thickener cells, in initial diactine stages of diactines (oscular and curved diactines, Fig. 2E). *SciTriactinin* is specific to triactine and tetractine thickener cells, and expression begins approximately with the separation of founder and thickener cells. In summary, of the seven biomineralization genes observed here, five are expressed in sclerocytes of all spicule types (*SciCA1*, SciCA2, *SciNCBT-like1, SciAE-like1* and *SciSpiculin*), and two are spicule type-specific (*SciDiactinin* and *SciTriactinin*). Furthermore, during initial spicule formation stages, the expression of founder and (prospective) thickener cells of one spicule type is identical. In later stages, the expression of the biomineralization genes changes, especially in the thickener cells, which no longer express the sclerocyte-specific CAs and SLC4 genes, but begin to produce the ARPs SciSpiculin and/or SciTriactinin. These observations are consistent with previous reports that in contrast to other species of calcareous sponges, the thickener cells of *S. ciliatum* do not appear to deposit additional calcite on the spicule^14^,^15^; therefore CA activity and bicarbonate transport are unnecessary in thickener cells. The missing thickening activity suggests another role for the thickener cells, which involves the expression of SciSpiculin and SciTriactinin (see below).

### Evolution of SLC4 and ARP proteins

**Figure 3:**
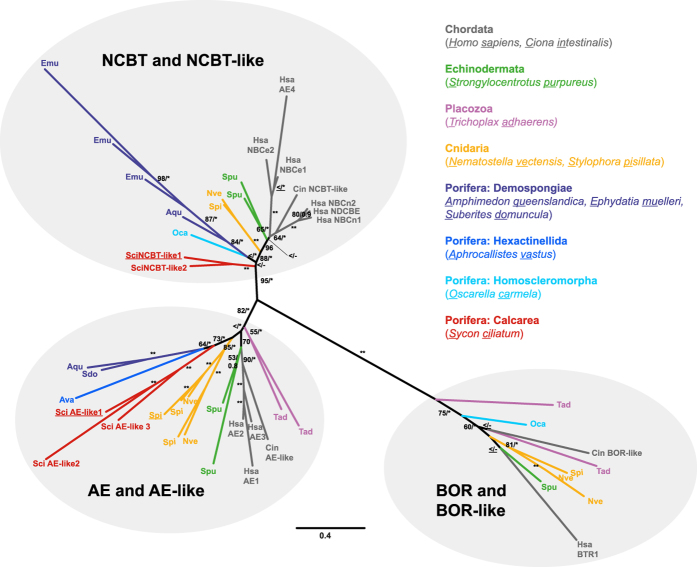
Phylogeny of SLC4 proteins. Maximum Likelihood tree (Phyml^48^, LG + I + G + F), bootstrap values (BS) of 200 replicates and posterior probability (PP) of Bayesian analysis (MrBayes^49^, LG + I + G + F, 5 million generations, burnin = 25% of sampled trees) are provided at the nodes (BS/PP; “*”= 100 BS or PP > 0.96; “** ” = BS of 100 and PP > 0.97; “ <=” support values below 50/0.5, “−” = node not present in Bayesian analysis), value on scale bar = substitutions/site. Biomineralization-specific proteins of *S. ciliatum* and *Stylophora pistillata* (SLC4*γ*) are underlined. The two biomineralization SLC4-proteins of *S. ciliatum* belong to the NCBT-like and the AE-like group, respectively.

**Figure 4:**
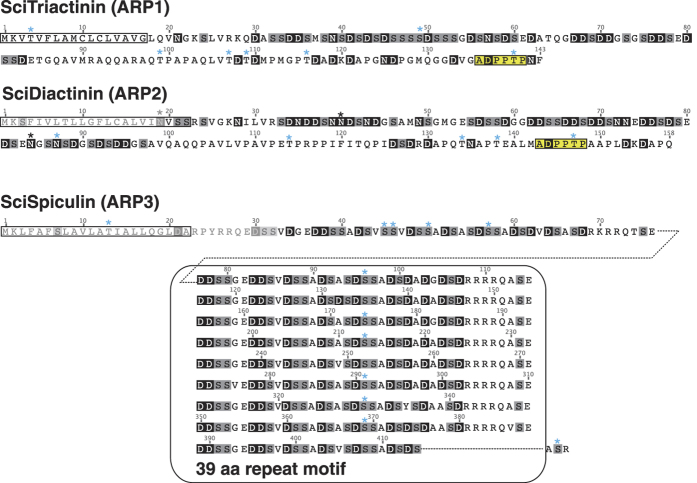
Amino acid sequences of the ARPs SciTriactinin (ARP1), SciDiactinin (ARP2) and SciSpiculin (ARP3). Aspartic acid and asparagine residues are highlighted by white letters on black background, serine by grey boxes. N-terminal signal peptides are marked by lined boxes, a short shared motif of *SciTriactinin* and *SciDiactinin* is marked by a yellow. Potential glycosylation sites are labelled with *(blue = O-linked glycosylation sites, black: N-linked glycosylation sites). Grey letters show parts that were not sequenced from cDNA due to position of the forward primers. For SciTriactinin and SciDiactinin, protein predictions from transcriptome data are presented, the SciSpiculin sequence is provided from combined clone sequence and transcriptome data.

Our phylogenetic analyses of SLC4 proteins revealed that sponge proteins occur in all three SLC4 groups (NCBT and NCBT-like, AE and AE-like, BOR and BOR-like, Fig. 3). SLC4 proteins of the BOR-like group are missing in calcareous sponges (and other sponges with the exception of the homoscleromorph *Oscarella,* Fig. 3). Because genomic data is not available for Hexactinellida, it cannot be excluded that additional SLC4-like transporters (NCBT and NCBT-like or BOR and BOR-like) are present in this sponge class. Nonetheless, our phylogenetic analyses (Fig. 3) confirm that sponges possess more SLC4-like transporters than only the previously reported AE-like protein of the demosponge *Suberites domuncula*^28^. In the clade “NCBT and NCBT-like” and “AE and AE-like”, many lineages, including calcareous sponges, show lineage-specific gene duplications: In addition to the two sclerocyte-specific SLC4 genes, of the three additional SLC4 proteins that are encoded in the *S. ciliatum* genome, one is placed in the NCBT and NCBT-like group (SciNCBT-like2), and is closest related to SiNCBT-like1 (Fig. 3). The other two were found to be of the AE and AE-like group and form a clade with SciAE-like1; within the clade, SciAE-like3 is the sister group to a clade of SciAE-like1 and SciAE-like2 (Fig. 3). Because *Sycon ciliatum* NCBT-like and AE-like BCTs are each monophyletic, a lineage-specific diversification of both SLC4 groups within Calcarea can be suggested. The biomineralizing SLC4*γ* from scleractinians also belongs to the AE-like SLC4-proteins, but is not especially closely related to the SciAE-like1 protein (Fig. 3). These transporters were likely independently recruited for the process of biomineralization in Calcarea and Scleractinia, possibly following lineage-specific duplications in both lineages. Similar observations were reported for the evolution of CAs^12^.

The evolution of the ARPs is more obscure. No conserved domains could be identified in the ARPs. BLAST searches against transcriptome of the closely related species *Sycon coactum*^29^ found one significant hit for SciTriactinin (*S. coactum* contig_18526), which has a sequence similarity of 47% and similar aspartic acid composition (*S. ciliatum*: 18.2%, *S. coactum* 17.2%) and serine contents (*S. ciliatum*: 16.8%, *S. coactum*: 18.6%) and may represent a true homolog of SciTriactinin (Suppl. Figure 2). It has an additional potential ORF that would encode 141 additional N-terminal amino acids if it would get translated (see legend of Suppl. Figure 2). This 141 amino acid sequence lacks any known protein domains and shows no homology by BLAST searches. An incomplete transcript (coding for 100 N-terminal amino acids) was found as significant BLAST-hit for SciSpiculin (*S. coactum* contig_22784). It contained two copies of a 30 amino acid repeat (Suppl. Figure 2). Possibly, the incompleteness is due to assembly problems and more repeats are present in the mature protein similar to the eight complete copies of the 39 amino acid motif in SciSpiculin. The similarity in the first 100 amino acid positions of SciSpiculin and the potential *S. coactum* homolog is 45%, their aspartic acid content is similar (*S. ciliatum*: 21.0%, *S. coactum*: 23.0%), while the *S. coactum* serine content is higher (*S. ciliatum*: 28.0%, *S. coactum*: 39%). No BLAST hits were found for SciDiactinin in *S. coactum*. BLAST searches of the ARPs (neglecting the signal peptides) against the transcriptome of the more distantly related calcareous sponge *Leucosolenia complicata*^22^ failed to provide any hits for any of the three ARPs (with maximum E-value cut-off of 10). Therefore, ARPs appear to be either evolving so fast that homology is obscured rapidly (e.g. in Diactinin even between very closely related species), or they represent lineage-specific innovations.

### Potential function of biomineralization genes

#### Potential function of SciAE-like1 and SciNCBT-like1

**Figure 5:**
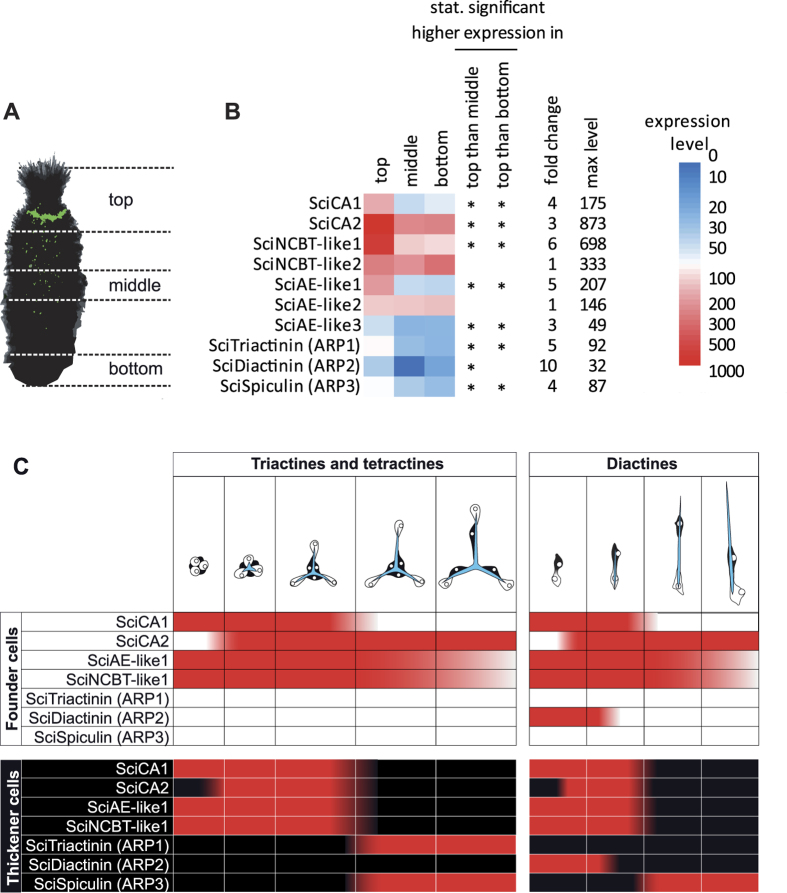
Spatial and temporal expression of seven biomineralization genes. (**A**) Schematic view of *S. ciliatum* body parts that were compared in the RNA-Seq analysis. The green colour depicts spicule formation, which is increased in apical regions, and was deduced from calcein-staining experiments^12^. (**B**) Expression levels of biomineralization genes and remaining SLC4 proteins in top, middle and bottom parts of *S. ciliatum*, the colour scale is from blue (lowest) through white (medium) to red (highest). Expression levels were calculated with expected_count from RSEM package^42^, normalized between datasets with the DESeq package^43^ and then log 10 transformed. Statistically significantly (padj ≤ 0.1) overexpressed genes in top vs. middle or top vs. bottom comparisons are marked by asterisks. (**C**) Summary of biomineralization gene expression in founder cells and (prospective) thickener cells of different spicule types, based on observations of the double ISH experiments. In both, tri- and tetractines on the one hand, and diactines on the other hand, the founder cells and prospective thickener cells show initially identical expression patterns. The most dramatic change of expression occurs in later stages in thickener cells, which of the seven genes only expresses *SciSpiculin* (all spicule types) and *SciTriactinin* (only triactine- and tetractine-specific thickener cells).

Although SLC4 proteins can be assigned to the groups AE-like, NCBT-like or BOR-like based on their phylogenetic affinities, the function and stoichiometry of transport of only a few members of each group are known, excluding for example sponge proteins^28^. Therefore, neither the direction nor the mode of transport (Na^+^-independent Cl- cotransport for SciAE-like1, or Na^+^-coupled for SciNCBT-like1) can be deduced for the two sclerocyte-specific SLC4 proteins in *S. ciliatum.* However, it is reasonable to assume that the proteins are involved in the guided transport of bicarbonate to the calcification site through the sclerocyte, i.e., trafficking bicarbonate from the mesohyl into the sclerocyte and/or trafficking bicarbonate that is formed within the sclerocyte through the activity of SciCA1 to the intercellular space of calcification^12^. This interconnected function of the sclerocyte-specific CAs and SLC4 proteins would also explain the striking similarity in their expression profiles in body parts (Fig. 5B).

#### Potential function ARPs

Acidic proteins with a high aspartic acid content have been found in the organic matrices of carbonate skeletons in many animals, including stony corals e.g., refs 30 and 31 and coralline demosponges^32^. Important functions of these proteins in the biomineralization process have been suggested^8^. For example, aspartic acid residues in these proteins have the ability to bind Ca^2+^ ions, and some can interact with specific crystal faces of growing biominerals, thereby influencing the crystal shape. Depending on the conditions, inhibition or promotion of crystallisation has been reported for acidic skeletal organic matrix (SOM) proteins^8^. The presence of Asx-rich-protein extracts of calcareous sponge spicules has been found to influence the shape of calcite crystal formation in *in vitro* experiments^20^. It has also been proposed that differences in crystal texture among spicule types of calcareous sponges are influenced by the acidic SOM proteins^33^; accordingly, specialized proteins were suggested to interact with specific crystal faces, inhibiting their growth, and thus influencing the preferred direction of crystal growth, which differed among triactines, curved diactines and oscular diactines. We therefore suggest that the spicule-type specific ARPs SciTriactinin and SciDiactinin are involved in the development of the different crystallographic growth patterns between diactines and triactines/tetractines.

Because *SciDiactinin* was expressed in the early stages of spicule formation, it presumably plays a role in the initial nucleation process of diactine spicules. However, our results cannot explain the previously reported differences between curved diactines and oscular diactines^33^. In contrast to the crystal texture of curved diactines, that of oscular diactines did not differ considerably from that of synthetic calcite, which was attributed to a lack or a low concentration of intraspicular proteins^33^. Yet, a difference in protein abundance might exist between the slender and curved diactines, potentially due, for example, to faster growth rates (about two times) of the former^12^. Also, species-specific differences between *S. ciliatum* and other species may exist: The ARPs are highly specific to *S. ciliatum*, and we found only two recognizable orthologs in the transcriptome of *Sycon coactum*, and none in the transcriptome of *Leucosolenia complicata*. The previous study^33^ mentioned above investigated a different species (*Sycon* sp. from the Mediterranean), but it is known that genus *Sycon* is polyphyletic, such that its member species may be only distantly related^34^.

Because *SciTriactinin* and *SciSpiculin* are only expressed in thickener cells in late spicule formation stages, they cannot act in the earlier stages. Observations on the organization of triactines from *Clathrina* sp. may be relevant: they possess a calcite core containing Asx-rich proteins, which is surrounded by a phase of amorphous calcium carbonate (ACC) stabilized by Glx-rich proteins and itself is covered by a thin calcitic sheath^20^. Although ACC has not been reported from other calcareous sponges^35^, it is difficult to detect, and it was speculated that stabilized ACC may be more widespread in calcareous sponges than is currently recognized^36^, potentially even as a transition stage in spicule maturation. Provided that the spicules in *S. ciliatum* show an identical organisation, SciTriactinin and SciSpiculin may be involved in the formation of the outermost thin calcitic sheath. In such a scenario, additional Asx-rich proteins from the calcitic core of triactines and tetractines of *S. ciliatum* could be expected, similar to the findings in *Clathrina* sp^20^. Mineralogical studies on the fine structure of newly formed spicules and the identification of additional ARPs, and Glx-rich proteins of a potential ACC layer could provide further insight.

## Conclusion

Spicule formation is a highly dynamic process that requires the concerted temporal regulation of gene expression in the sclerocytes involved to build the complex architecture of the calcareous sponge skeleton. The expression of the seven biomineralization genes studied here in the prospective founder and thickener cells of each spicule type is identical in the initial stages of spicule formation. In later stages of spicule formation, expression of founder and thickener cells differentiate from each other. This observation is consistent with the fact that each thickener and founder cell pair develops from a single precursor cell with subsequent spatio-temporal diversification^15^. Of the seven biomineralization genes analysed, the two biomineralizing CAs and the two biomineralizing SLC4 genes and the ARP *SciSpiculin (ARP3*) provide a common genetic ground pattern for the formation of all spicule types of *S. ciliatum*. In contrast, the ARPs *SciDiactinin (ARP2*) and *SciTriactinin (ARP1*) are spicule type-specific modifications in the genetic biomineralization toolkit and present evidence for genetic determination of biomineral shape in calcareous sponges. Our results highlight that genetic control over the biomineralization is essential in the formation of different biomineral shapes as observed even in such simple biominerals as calcitic sponge spicules, which are formed by only a few cells.

## Methods

### Identification of biomineralization genes

*Sycon ciliatum* sponges were collected in Norway, tissue fixed for RNA extraction and RNA *in situ* hybridization as described before^12^,^22^,^24^. Previous studies provided transcriptomes of different life-cycle stages and body parts and provided lists of genes with higher expression in apical body parts^22^,^24^, in which biomineralization is increased^12^. From this list, two bicarbonate transporters of the SLC4 family were identified. ARPs were identified by selecting apically overexpressed genes with Asx- contents larger than 20% and with a signal peptide. While *SciTriactinin (ARP1*) and *SciDiactinin (ARP2*) were complete transcripts, the transcriptome assembly of *SciSpiculin (ARP3*) did not yield the C-terminal stop codon, probably due to the presence of a 117 bp repeat motif (coding for 39 amino acids), and we therefore identified the corresponding ORF on the genomic scaffold 29508^22^ to design 5′ primers.

### Cloning, sequencing and sequence analysis

Primers (Suppl. Table 1) for each of the target genes were designed using the primer3 as implemented in Geneious R8 (http://www.geneious.com)^37^. *SciTriactinin* and *SciDiactinin* reverse primers were designed to introduce a T7 recognition site for RNA antisense probe generation from PCR products. A pool of cDNA from different life stages was used as template for PCRs. PCR-products of all templates were cloned into the pCR4 vector (Invitrogen), clones were prepared for sequencing with vector-specific primers using the BigDye Terminator sequencing kit v.3.1 (Applied Biosystems). Bidirectional sequencing was performed at the Sequencing Service at the LMU Biozentrum on an ABI 3730 capillary sequencer (Applied Biosystems). Forward and reverse sequences were assembled in Geneious R8 (http://www.geneious.com)^37^. All sequences have been submitted to the European Nucleotide Archive (accession codes LT674110- LT674121, http://www.ebi.ac.uk/ena/data/view/LT674110-LT674121). Alignments of genomic and amplified sequences are available in the Open Data LMU repository (http://dx.doi.org/10.5282/ubm/data.97). Cloning of *SciTriactinin (ARP1*) yielded two versions of which one had a six base pair (two amino acid) insertion compared to the transcriptome sequence (scigt017205). The sequenced *SciDiactinin (ARP2*) fragment did not cover the complete 5′ coding region of the gene. For further analyses, the predicted gene sequences from the genome were used. Sequencing of *SciSpiculin* (ARP3) cDNA revealed three additional 117 bp direct repeats compared to the genomic sequence. We believe that the genomic assembly probably failed to assemble the 8 × 117 bp repeat region of the gene, which may also be the reason for the incompleteness of the transcriptomic sequence (see above). For further analyses, the clone sequence was complemented with the 5′ end of the transcriptomic and genomic sequence, which was not amplified with our primers. Amino acid composition and isoelectric point of ARPs were determined in Geneious R8 (http://www.geneious.com)^37^. Signal peptides of ARPs were detected with signalP 4.1^38^, potential glycosylation sites were predicted with GlycoEP (http://www.imtech.res.in/raghava/glycoep)^27^. BLAST searches^39^ against GenBank databases and searches in pfam^40^,^41^ were conducted, but for the ARPs yielded no significant similarities to known proteins or domains. BLAST was also used to identify ARPs in the transcriptome of *Sycon coactum* (https://era.library.ualberta.ca/files/bjh343s467#.WE53UKKLS1s)^29^. Bicarbonate transporters SciNCBT-like1 and Sci-AE-like1 were unambiguously homologous to other SLC4 proteins, to which they could be aligned (see below).

### RNA *in situ* hybridization and RNA-Seq

Antisense RNA probes of all five genes were generated by *in vitro* transcription using T7 or T3 RNA polymerase and plasmids or PCR products (for *SciTriactinin, SciDiactinin*) with introduced T7 sites in the reverse primers (Suppl. Table 1). Probes were labeled using the digoxigenin (DIG) or fluorescein RNA labelling kit (Roche). RNA antisense probes for *SciCA1* and *SciCA2* were available from a previous study^12^. Fixed tissues of *S. ciliatum* (small sponges or parts of larger sponges) were used in ISH experiments, which were performed according to previously published protocols^12^,^21^. For double ISH, two probes labelled with either DIG or fluorescein were applied, and the first probe was detected with NBT/BCIP and the second with Fast Red (Roche). Whole mount ISH experiments were documented with Leica M165F or Leica DMLB microscope. To increase the depth of field, multi-focus images were combined with Helicon Focus 4.2.9 (HeliconSoft).

In detail RNA-Seq analysis of the expression of the seven biomineralization genes and the remaining SLC4 genes was performed using existing transcriptomic RNA-seq datasets from top, middle and bottom body section of *S. ciliatum* sponges^22^ available at ArrayExpress (http://www.ebi.ac.uk/arrayexpress) under accession number E-MTAB-2430. Expression levels were calculated with expected_count from RSEM package^42^, normalized between datasets with the DESeq package^43^ and then log 10 transformed. Statistically significantly (padj ≤ 0.1) overexpression of genes was determined in comparisons top vs. middle or top vs. bottom.

### Phylogenetic analysis of SCL4 proteins

Additional SCL4 proteins of *S. ciliatum* and other phyla were identified by BLAST^39^ from available transcriptomic or genomic data (Suppl. Table 2). Protein sequences were aligned with MUSCLE^44^ implemented in Seaview^45^. Gblocks^46^ was used to select conserved sites suitable for the phylogenetic analyses. The best fitting model for Maximum Likelihood (ML) analysis and Bayesian inference (LG + I + G + F) was determined under the Akaike Information Criterion (AIC) with Prottest3^47^. ML likelihood analysis including a 200 replicate bootstrap analysis was performed with PhyML 3^48^. Bayesian inference was conducted in MrBayes 3.2.6^49^ (5 million generations, sampling every 200th tree and discarding the first 25% of sampled trees as burnin to calculate the consensus tree). Sufficient parameter sampling of the analysis was confirmed by inspection of the parameter files in tracer v1.6 (http://tree.bio.ed.ac.uk/software/tracer/). The SLC4 alignment (including sequence identifiers and information about sites included in the analyses) is available via the Open Data LMU repository (http://dx.doi.org/10.5282/ubm/data.97).

## Additional Information

**Accession codes**: Sequences of mRNA PCR products/clones have been submitted to the European Nucleotide Archive (accession codes LT674110- LT674121, http://www.ebi.ac.uk/ena/data/view/LT674110-LT674121). The RNA-seq dataset used for this study is available at ArrayExpress (http://www.ebi.ac.uk/arrayexpress) under accession number E-MTAB-2430).

**How to cite this article:** Voigt, O. *et al*. Spicule formation in calcareous sponges: Coordinated expression of biomineralization genes and spicule-type specific genes. *Sci. Rep.* **7**, 45658; doi: 10.1038/srep45658 (2017).

**Publisher's note:** Springer Nature remains neutral with regard to jurisdictional claims in published maps and institutional affiliations.

## Supplementary Material

Supplementary Information
