# Supplementary material for: Spicule formation in calcareous sponges: Coordinated expression of biomineralization genes and spicule-type specific genes
Source: Sci Rep. 2017 Apr 13;7:45658. doi: 10.1038/srep45658 (PMC5390275; doi:10.1038/srep45658)
Supplement: Supplementary Information [file srep45658-s1.pdf]

Supplementary Information for:

Spicule formation in calcareous sponges: Coordinated expression of biomineralization genes and spicule-type specific genes

Oliver Voigt, Maja Adamska, Marcin Adamski, André Kittelmann, Lukardis Wencker, Gert Wörheide

Supplementary Table 1: Primer sequences.

Primer	Sequence (5'-3')
SciAE-like1 fw	GCTCAAGTTCGAAGAGGAGG
SciAE-like1 rv	GCAGTAGAAGGTCCTTGTCG
SciNCBT-like1 fw	CAACCTGAGTCCGGTACATG
SciNCBT-like1 rv	CTCGGTGAAGCGAGTAAACA
Triactinin (ARP1) fw	CAAGTTGTTCTGAACGCGTC
Triactinin (ARP1) rv incl. T7 (underlined)	GAAATTAATACGACTCACTATAGGCAGGTGCTTACAGGAGAACG
Diactinin (ARP2)	GTTGTTTTGGCCTTGTTGGG
Diactinin (ARP2) rv incl. T7 (underlined)	GAAATTAATACGACTCACTATAGGTGCAGATTGAGGATGAGTGC
Spiculin (ARP3) fw	GTGTTGATGGCGAGGATGAT
Spiculin (ARP3) rv	TTATCTGCTTGCTGAGTCGG

Supplementary Table 2: Sources of SLC4 protein sequences (G= protein sequences derived from genomic data, T= protein sequences derived from transcriptomic data).

Phylum	Organism	Transcriptome/ Genome	Data version	Source
Chordata	Homo sapiens	G	CCDS	http://www.ncbi.nlm.nih.gov
Cnidaria (Anthozoa)	*Nematostella vectensis*	G	N.vectensis v1.0 models	http://genome.jgi.doe.gov/
Cnidaria (Anthozoa)	*Stylophora pistillata*	T	n.a.	(Zoccola et al. 2015)
Echinodermata	*Strongylocentrotus purpuratus*	G	protome	http://www.metazome.net/
Placozoa	*Trichoplax adhaerens*	G	Triad1_best_transcripts	http://genome.jgi.doe.gov/
Porifera (Calcarea)	*Sycon ciliatum*	T	SCIL_T-PEP_130802	http://compagen.zoologie.uni-kiel.de/
Porifera (Demospongiae)	*Ephydatia muelleri*	T	EMUE_T-PEP_130911	http://compagen.zoologie.uni-kiel.de/
Porifera (Demospongiae)	*Amphimedon queenslandica*	G	Aqu1	http://www.ncbi.nlm.nih.gov
Porifera (Hexactinellida)	*Aphrocallistes vastus*	T	PRJNA225584	http://www.ncbi.nlm.nih.gov/bioproject/
Porifera (Homoscleromorpha)	*Oscarella carmela*	G	OCAR_G-PEP_120614	http://compagen.zoologie.uni-kiel.de/

Zoccola, Didier, Philippe Ganot, Anthony Bertucci, et al. 2015. "Bicarbonate transporters in corals point towards a key step in the evolution of cnidarian calcification." *Scientific Reports* 5 (June): 9983.

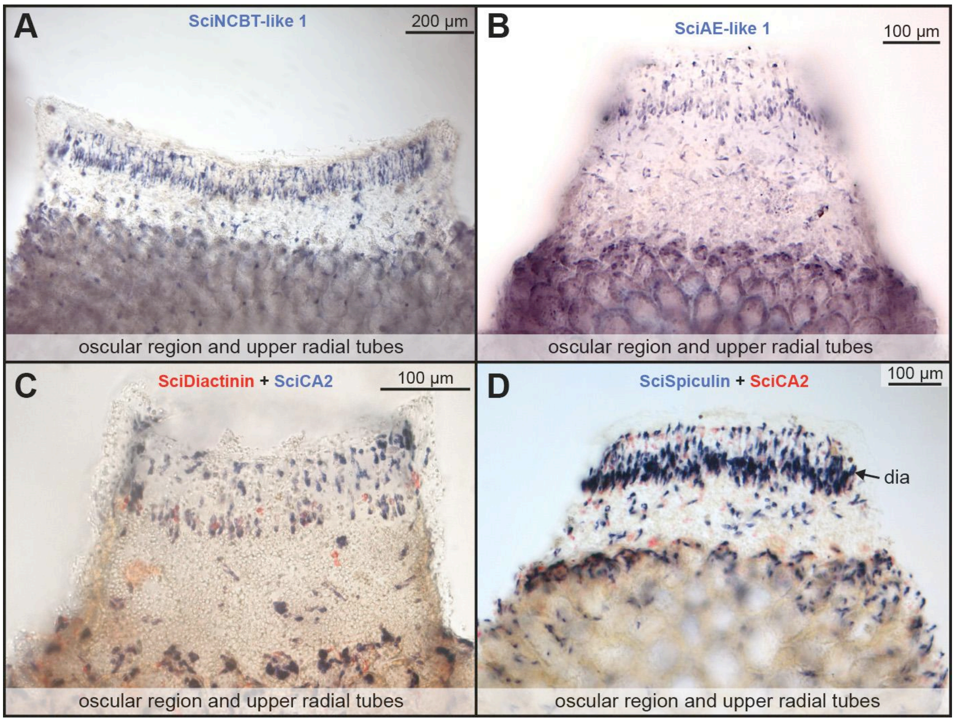

Supplementary Figure 1: Expression of biomineralization genes in the oscular region and the upper radial tubes in small *Sycon ciliatum* sponges: (A) *SciNCBT-like1*, (B) *SciAE-like1*, (C) *SciDiactinin* and *SciCA2*, (D) *SciSpiculin* and *SciCA2*.

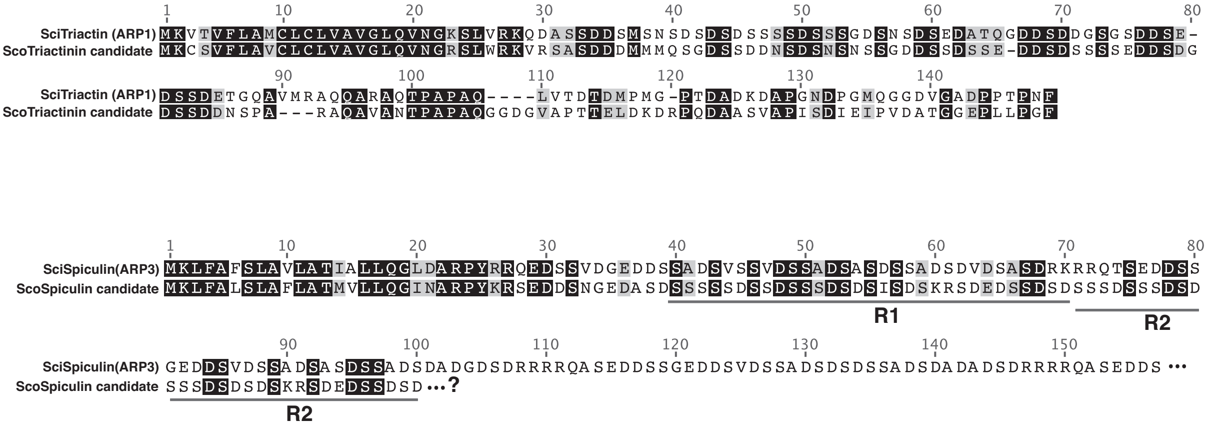

Supplementary Figure 2: Alignment of SciTriactinin (above), partial SciSpiculin (below) and potential homologs of the closely related species *Sycon coactum* (Sco). The shading depicts similarity.
Top: ScoTriactinin candidate (contig_18526) contained a longer 5' ORF, which would translate to additional 141 amino acids:
MNIDARPIVVRGHCGLISSISCCSTAIRRMAPSVRNANCGLQLVNTSGTFIQWAAEAEASWNEGADSVGRQSSEVTALTXTDGDIQLASLQLGSVSIKELSFVSITISEATPLTTKGKFESLLWTGQFFFLSDSSSKQAVK.
Bottom: ScoSpiculin candidate (contig_22784) is incomplete at the C-terminus, possibly due to additional repeats of a 30 amino acid motif (R1 and R2) that may have hampered a correct assembly of the whole transcript.
